# Targeting the NAD^+^ salvage pathway suppresses APC mutation-driven colorectal cancer growth and Wnt/β-catenin signaling via increasing Axin level

**DOI:** 10.1186/s12964-020-0513-5

**Published:** 2020-01-31

**Authors:** Chenyang Ye, Lina Qi, Xiaofen Li, Ji Wang, Jiekai Yu, Biting Zhou, Cheng Guo, Jiani Chen, Shu Zheng

**Affiliations:** 1 Cancer Institute (Key Laboratory of Cancer Prevention and Intervention, China National Ministry of Education), the Second Affiliated Hospital, School of Medicine, Zhejiang University, Zhejiang, 310009 Hangzhou China; 2 Department of Abdominal Oncology, West China Hospital, Sichuan University, Chengdu, Sichuan China; 3 Department of Surgical Oncology, Sir Run Run Shaw Hospital, School of Medicine, Zhejiang University, Zhejiang, 310016 Hangzhou China; 4 Biomedical Research Center and Key Laboratory of Biotherapy of Zhejiang Province, Zhejiang, 310016 Hangzhou China; 5 Reseach Center for Air Pollution and Health, School of Medicine, Zhejiang University, Zhejiang, 310009 Hangzhou China

**Keywords:** NAD^+^, NAMPT, FK866, Colorectal cancer tumors, Proliferation, Wnt/β-catenin

## Abstract

**Background:**

The role and mechanism of the nicotinamide adenine dinucleotide (NAD^+^) salvage pathway in cancer cell proliferation is poorly understood. Nicotinamide phosphoribosyltransferase (NAMPT), which converts nicotinamide into NAD^+^, is the rate-limiting enzyme in the NAD^+^ salvage pathway. Here, we assessed the role of NAMPT in the proliferation of colorectal cancer.

**Methods:**

Real-time PCR, immunohistochemistry, western blotting, and analyses of datasets from Oncomine and Gene Expression Omnibus were conducted to assess the expression of NAMPT at the mRNA and protein levels in colorectal cancer. The Kaplan Meier plotter online tool was used to evaluate the prognostic role of NAMPT. Knockdown of NAMPT was performed to assess the role of NAMPT in colorectal cancer cell proliferation and tumorigenesis both in vitro and in vivo. Overexpression of NAMPT was used to evaluate impact of NAMPT on colorectal cancer cell proliferation in vitro. NAD^+^ quantitation, immunofluorescence, dual luciferase assay and western blot were used to explore the mechanism of colorectal cancer proliferation. Transwell migration and invasion assays were conducted to assess the role of NAMPT in cell migration and invasion abilities of colorectal cancer cells.

**Results:**

Our study indicated that the inhibition of NAMPT decreased proliferation capacity of colorectal cancer cells both in vitro and in vivo. Conversely, overexpression of NAMPT could promote cell proliferation in vitro. NAMPT inhibition induced β-catenin degradation by increasing Axin expression levels; this resulted in the inhibition of Wnt/β-catenin signaling and cell proliferation in colorectal cancer. The addition of nicotinamide mononucleotide, the enzymatic product of NAMPT, effectively reversed β-catenin protein degradation and inhibited growth. Similarly, the knockdown of Axin also decreased the cell death induced by the inhibition of NAMPT. In addition, we showed that colorectal cancer tissues harbored significantly higher levels of NAMPT than the levels harbored by paired normal tissues, especially in colorectal cancer stages I and II. And the overexpression of NAMPT was associated with unfavorable survival results.

**Conclusions:**

Our findings reveal that NAMPT plays an important role in colorectal cancer proliferation via Wnt/β-catenin pathway, which could have vital implications for the diagnosis, prognosis and treatment of colorectal cancer.

## Background

Colorectal cancer (CRC) is the third most common diagnosed and death-causing cancer in both men and women in the United States in 2019 [1]. Despite improvements to diagnostic techniques and therapeutics over the last two decades, less than 20% of newly-diagnosed metastatic colorectal cancer patients survive more than 5 years [2]. This situation highlights the pressing need to find better biomarkers for early stage detection and optimal therapeutic targets for the control of CRC progression.

CRC cells require specific adaptations of cellular metabolism to support high proliferative capacity [3, 4]. Since NAD^+^ is a vital component that participates in multiple aspects of cellular metabolism, a higher basal NAD^+^ turnover is required to meet enhanced metabolic requirements of CRC cells [5–7]. In cancer cells setting, the biosynthesis of NAD^+^ is mainly via the salvage pathway from nicotinamide (NAM), nicotinic acid (NA) and NAM riboside (NR) rather than via the de novo pathway from tryptophan [6]. Nicotinamide phosphoribosyltransferase (NAMPT), also known as visfatin or pre-B cell enhancing factor, is the rate-limiting enzyme in the NAD^+^ salvage pathway. NAMPT converts 5-phosphoribosyl-1-pyrophosphate (PRPP) and nicotinamide (NAM) into nicotinamide mononucleotide (NMN) [8–10]. Then, NMN is converted into NAD^+^ by NAM mononucleotide adenylyltransferases [11, 12]. In addition to important effects on cellular metabolism, NAMPT has also been found to participate in a variety of oncogenic cellular processes, including tumor proliferation, apoptosis, metastasis, inflammation, DNA repair and angiogenesis [13–19]. NAMPT also serves as a biomarker and prognostic indicator in cancer [11, 14]. However, the function and mechanism of NAMPT in CRC remains to be clarified.

According to the comprehensive and integrated analysis of the human CRC genome, the Wnt/β-catenin signaling pathway is one of the most important pathways in the initiation and progression of CRC [20]. Currently, the Wnt/β-catenin signaling pathway lacks druggable molecular targets, which has hampered the development of therapeutic drugs targeting this pathway. It is unknown whether NAMPT regulates colorectal cancer proliferation through Wnt/β-catenin signaling pathway. If NAMPT could regulate Wnt/β-catenin pathway, then targeting NAMPT such as using its inhibitor FK866 could be a potential therapeutic option to retard tumor growth.

In this study, we discovered a novel mechanism of the regulation of CRC proliferation by NAMPT. NAMPT was found to be significantly overexpressed in the early stages of CRC (stages I and II) through proteomics and expression microarrays compared to the expression in adjacent normal tissues. Thus, we presumed that NAMPT might contribute to proliferation. Next, in vitro and in vivo experiments confirmed our hypothesis. Interestingly, we found that the Wnt/β-catenin pathway contributed to this process. Consequently, NAMPT regulated cell proliferation by activating the Wnt/β-catenin pathway and could be used as a biomarker for early stage CRC screening and a drug target after further research.

## Methods

### Oncomine database analysis

The expression levels of *NAMPT* gene in CRC and adjacent normal tissues were analyzed using Oncomine, a cancer microarray database and web-based data mining platform from genome-wide expression analyses [21, 22]. The mRNA expression level in cancer tissues compared to the noncancerous control tissues was obtained as the parameters of *p* value < 0.001, fold change > 2, and gene ranking in the top 10%.

### Gene expression from TCGA

The Cancer Genome Atlas (TCGA) gene expression profile of NAMPT was obtained from a web portal UALCAN [23]. In UALCAN (http://ualcan.path.uab.edu/), the clinical data for patients with colon cancer and Level3 TCGA RNA-seq data (including raw read count and scaled estimate for each sample) for primary tumors and matched normal samples were downloaded using TCGA assembler [24]. For each gene, transcript per million values were obtained by multiplying the scaled estimate by 1,000,000. Boxplots were generated by use of R (https://cran.r-project.org/).

### The Kaplan-Meier plotter database

The prognostic merit of gene mRNA expression was appraised by an online database, Kaplan-Meier Plotter (www.kmplot.com) [25], which included gene expression data and survival information of clinical CRC patients from Gene Expression Omnibus (GEO) and the Cancer Genome Atlas (TCGA) databases. To analyze the overall survival (OS) and relapse free survival (RFS) of patients with CRC patient samples were split into two groups by median expression (high vs. low expression) and assessed by a Kaplan-Meier survival plot, with the hazard ratio (HR) with 95% confidence intervals (CI) and log-rank *p* value.

### Sample collection and patient characteristics

For immunohistochemistry (IHC) analysis, the CRC tissue microarray (TMA) including paired CRC and adjacent normal tissues surgically collected from 50 patients, were collected from Wuhan Servicebio technology company. For mRNA and protein analyses, 20 pairs of CRC and adjacent normal tissues were surgically obtained from the Second Affiliated Hospital, Zhejiang University School of Medicine, and frozen at − 80 °C. Written, informed consent was obtained from each patient. The Ethics Committee of the Second Affiliated Hospital at Zhejiang University, School of Medicine approved this study.

### IHC staining and semiquantitative analysis

CRC TMA was heated, deparaffinized and treated with citrate antigen repair buffer (pH 6.0) for antigen repair with 3% hydrogen peroxide to block endogenous peroxidase activity and 3% BSA for serum blocking. The TMA was incubated with an anti-NAMPT primary antibody (1:250, Abcam, ab45890) and with the coordinating secondary antibody. Staining was displayed with DAKO DBA solution. Harris hematoxylin was used to restain the nucleus, and TMA was dehydrated by alcohol. The stained TMA was scanned using the Pannoramic Midi and was analyzed using the Pannoramic Viewer (3D Histech) and Quant center. The software automatically identified and scored all brown staining on the tissue section as follows: dark brown = 3, brown yellow = 2, light yellow = 1, blue nucleus = 0, and the software evaluated the extent of stained cells (0–5% = 0; 5–25% = 1; 26–50% = 2; 51–75% = 3 and 76–100% = 4). The final score was determined by multiplying the intensity score and the score for the extent of stained cells, generating a score that ranged from 0 to 12. The staining results were categorized into negative (score 0; −), low (score 1–4; +), moderate (score 5–8; ++), and high (score 9–12; +++). The results were evaluated by two independent pathologists.

### Subcellular protein fractionation and western blotting analysis

Total protein extracts were prepared using RIPA buffer (Beyotime) in the presence of a proteinase inhibitor mixture (Roche Applied Science). Nuclear and cytoplasmic protein extracts were prepared using a Nuclear and Cytoplasmic Protein Extraction Kit (Beyotime). Protein extracted from the cells or from fresh-frozen tissues was loaded and separated by 10% SDS-polyacrylamide gel electrophoresis (SDS-PAGE). Then, the proteins were transferred onto polyvinylidene fluoride (PVDF) membranes by electrophoresis and were incubated with the primary antibodies. Immunoreactive bands were detected by chemiluminescence using corresponding horseradish peroxidase (HRP)-conjugated secondary antibodies and enhanced chemiluminescence (ECL) detection reagents. Gray intensity analysis of the western blot images was conducted using ImageJ software. Then, the relative protein abundance was determined. The primary antibodies used for western blot include the following: anti-NAMPT (Cell Signaling Technology, # 61122), anti-GAPDH (Cell Signaling Technology, # 5174), anti-β-catenin (Cell Signaling Technology, # 8480), anti-cyclin D1 (Cell Signaling Technology, # 2978), and anti-Axin (Cell Signaling Technology, # 2087).

### Quantitative reverse transcription-polymerase chain reaction (qRT-PCR)

Total RNA was extracted from fresh-frozen CRC tissues or CRC cells. The Takara PrimeScript™ RT Master Mix Kit (Takara, RR036Q) was used for reverse transcription. The SYBR Premix Ex Taq II Kit (Takara, RR820A) and Applied Biosystems 7500 Fast Real-Time PCR System were applied for real-time PCR analysis. Experiments were carried out in triplicate, and GAPDH was used as the loading control. The forward primer sequence of NAMPT was AATGTTCTCTTCACGGTGGAAAA (5′ to 3′), and the reverse primer sequence was ACTGTGATTGGATACCAGGACT (5′ to 3′). The forward primer sequence of GAPDH was ATCCCATCACCATCTTCCAG (5′ to 3′), and the reverse primer sequence was TGAGTCCTTCCACGATACCA (5′ to 3′). The ΔΔCt method was applied to evaluate the mRNA relative expression level.

### Cell culture

The LoVo, SW480, SW620, RKO, HCT116, HT29 and DLD1 cell lines were purchased from ATCC and were cultured in DMEM at 37 °C in 5% CO_2_. The culture medium was supplemented with 10% FBS (HyClone), 100 U/mL penicillin and 100 mg/mL streptomycin.

### Treatment of NMN or FK866

The inhibitor FK866 (cat # S2799) and NMN (cat # S5259) were obtained from Selleck Chemicals. LoVo and RKO cells were seeded into 96-well culture plates at 1000 cells/well and were allowed to attach for 24 h in an incubator before treatment with FK866 or NMN.

### NAD^+^ quantitation

Measurement of NAD^+^ was conducted using SIGMA NAD/NADH Quantitation kit (MAK037) following the manufacturer protocol. Briefly, 1 × 10^5^ cells harvested for total NAD extraction and quantification. The NAD Cycling Enzyme Mix recognizes NAD^+^ and NADH instead of NADP or NADPH. Concentration of NAD total (NAD^+^ + NADH) was measured by absorbance at OD 450 nm. The NAD^+^ concentration was calculated by subtracting the NADH values from NADtotal.

### Stable gene knockdown and overexpression using lentiviral gene delivery

According to published papers and results from the blast tool in the NCBI database, we used the following target sequences: NAMPT (5-′ GAGTGTTACTGGCTTACAA-3′) [13], Axin (5′- GAGGAAGAAAAGAGAGCCA-3′) [26], and a scrambled sequence (5′- GAGTGTTACGGGGTTCCAG-3′). These sequences were cloned into GV248/GV307 vectors (GeneChem, Shanghai, China). All plasmids were transfected into 293 T cells together with the Lentivector Expression System (GeneChem, Shanghai, China) to produce lentivirus. These specific shRNAs were packaged into lentiviruses by GeneChem Inc. For NAMPT overexpression, the published method was used [13]. For the infection, the target cells were cultured at 100,000 cells per well in 6-well plates, cocultured with 2.5 × 106 Tu virus in the presence of 5 mg/ml polybrene and standard medium for 13 h, and then cultured with fresh medium. After 72 h of transfection, the cells were selected via incubating with 1 μg/ml puromycin for 1 week. We utilized Western blot analyses to confirm the expression of the target genes.

### Cell viability analysis

Cell Counting Kit-8 (CCK-8, Dojindo, Japan, CK04)) was utilized to evaluate the cell proliferation. The experiments were performed according to the manufacturer’s protocol. Briefly, 1 × 10^3^ cells were seeded into a 96-well plate containing 100 μL of completed culture medium per well that was incubated in a 37 °C incubator. Culture medium was used as a blank control. The cell proliferation was evaluated every day for 5 days after plating. CCK-8 solution (10 μL) was added to each well, and then, the plate was incubated with the cells in the 37 °C incubator for 3 h. An optimal density (OD) value of 450 nm was used to measure the cell proliferation. The mean and SD were calculated from three independent assays.

### Cell colony formation assay

Cell colony formation experiments were performed to reflect anchorage-independent cell growth. Approximately 1000 cells were seeded into a 6-well plate containing complete culture medium that was incubated in a 37 °C incubator. Colonies consisting of more than 50 cells after 2 weeks were counted.

### Immunofluorescence assay

The cells seeded onto glass slides were fixed in 4% formaldehyde for 10 min and blocked with PBS-T (PBS + 0.1% Tween 20) containing 1% BSA and 0.1% Triton X-100 for 1 h at room temperature. Next, anti-visfatin (Abcam, ab45890) and anti-Ki-67 (CST, Mouse mAb #9449) antibodies were diluted in blocking buffer according to the manufacturer’s recommendation and were incubated with cells at 4 °C overnight. After washing with PBS-T three times for 5 min each, Alexa Fluor 488-labeled (Abcam, ab150073) and Alexa Fluor 568-labeled (Abcam, ab175472) secondary antibodies were diluted in blocking buffer according to the manufacturer’s recommendation and were incubated with the cells at room temperature for 1 h. Before adding DAPI, the slides were washed with PBS-T three times for 5 min each. A confocal microscope (Zeiss) was used for taking photographs.

### Flow cytometry for apoptosis analysis

Trypsin (0.25%) without EDTA was used to harvest cells (1 × 10^6^–10^7^ cells) in a 6-well culture flask (in triplicate for experiments). The Annexin V-FITC/PI Apoptosis Kit used in this experiment was manufactured by Lianke (China, Cat#: AP101–30-kit). The collected cells were washed twice with PBS and were resuspended in 500 μl pre-cooled 1x Binding Buffer. Then, 5 μl Annexin V-FITC and 10 μl PI were added to the cell suspension and vortexed softly. The cells were incubated for 5 min at room temperature in the dark. The cells were analyzed by flow cytometry (FACSCanto II; BD Biosciences, San Jose, CA, USA) directly without washing. The cells treated with 0.1% DMSO were used for parameter adjustment.

### Dual luciferase assay

Cells were seeded in 24-well plates and transfected the following day with a total of 0.6 μg of DNA/well (0.5 μg of TOPFLASH, and 0.1 μg of thymidine kinase promoter Renilla). The lysates were collected 48 h post-transfection and used with the dual luciferase reporter system (Promega). Firefly and Renilla luciferase activity was measured in a luminometer. Normalized data expressed in relative luciferase units was averaged from triplicate assays, and the error bars reflect the standard deviations.

### Transwell migration and invasion assays

For migration assays, cells were seeded in the upper chamber of a 6.5-mm Transwell with 8.0-μm pore polycarbonate membrane inserts (Corning, USA) in 200 μl of serum-free medium containing for 48 h at 37 °C. Complete growth medium with 20% FBS was placed in the bottom compartment as a chemoattractant. After incubation, nonmigrated cells were wiped off from the upper surface using cotton swabs. The migrated cells on the lower surface were fixed with 4% paraformaldehyde and stained using crystal violet. Five random fields of migrated cells were imaged at a magnification of 200×; migrated cells were quantified using ImageJ analysis software. For invasion assays, cells were seeded in the upper chamber of a 6.5-mm Transwell with 8.0-μm PET membrane precoated with Matrigel (Corning, USA) in 200 μl of serum-free medium for 72 h at 37 °C. Invaded cells were fixed, stained, imaged, and counted as described above.

### Xenografts in nude mice

Animal experiments were conducted according to the Animal Study Guidelines of Zhejiang University. Five-week-old female nude mice (BALB/C) were used for the animal study. To construct the subcutaneous tumor xenograft mouse model, 5 × 10^6^ tumor cells were injected subcutaneously at the costal margin. The size of the xenograft tumor was measured every 3 days. The mice were sacrificed 25 days later, and the subcutaneous xenograft tumors were dissected and weighed. Portions were immediately frozen in liquid nitrogen for the following extraction of protein or were fixed in 10% buffered formalin for immunohistochemistry staining.

### Statistical analysis

SPSS Statistics 23.0 (IBM, Armonk, NY, USA) was used to conduct statistical analyses. The statistical tests were two-sided, and *p* < 0.05 was considered statistically significant. Two-tailed Student’s t-tests were used to compare quantitative data.

## Results

### Increased expression of NAMPT in CRC

**Fig. 1:**
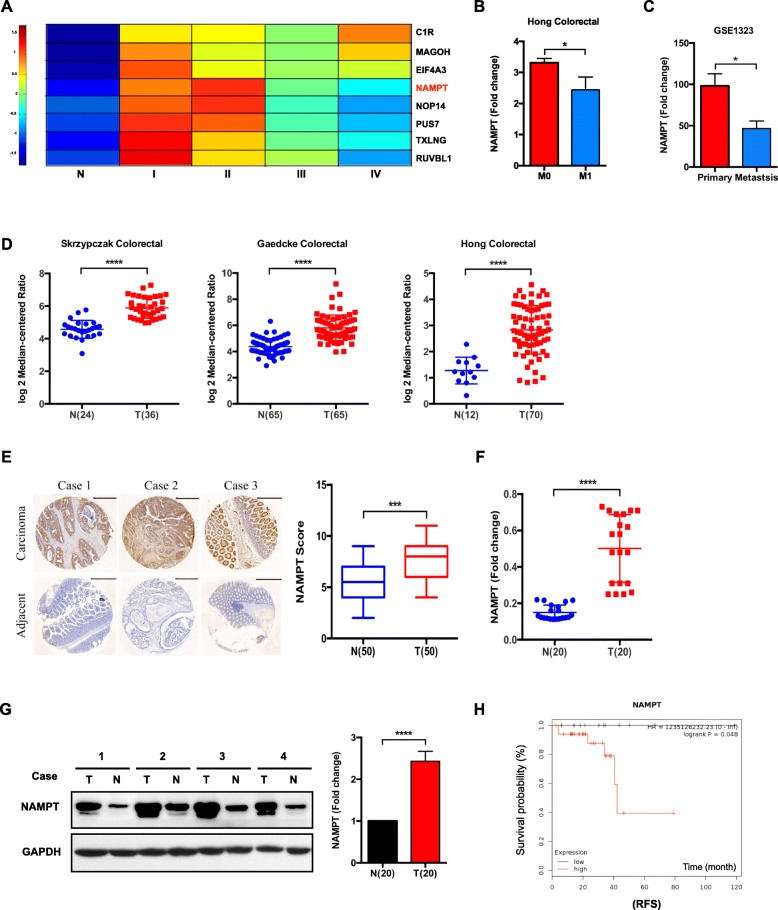
NAMPT expression and prognostic analysis in human colorectal cancer (CRC). **a** The expression analysis of the proteogenomic study showed NAMPT expression levels in four stages of CRC and adjacent normal tissues. **b** NAMPT mRNA levels in M0 stage compared with M1 stage using CRC patient tissues. Data were obtained from the Oncomine Cancer Microarray Database using the dataset “Hong Colorectal”. **c** NAMPT mRNA expression statuses in primary CRC cell line (SW480) and metastatic cell line (SW620) were obtained from GSE1323. SW480 and SW620 are CRC cell lines derived from a primary tumor and a corresponding metastasis lesion from the same patient. **d** NAMPT mRNA levels in CRC patient tissues compared to nontumorous tissues. Data were obtained from the Oncomine Cancer Microarray Database using the datasets “Gaedcke Colorectal”, “Skrzypczak Colorectal” and “Hong Colorectal”. **e** Immunohistological (IHC) analysis of NAMPT protein expression in human CRC and corresponding normal tissues using tissue microarrays. Exemplary paired samples are presented. A summarized quantification of the IHC score is depicted. **f** NAMPT mRNA expression levels (qRT-PCR analysis) in human CRC tissues compared to those in paired normal tissues. **g** The relative NAMPT protein intensity in the CRC tissues compared to that in the adjacent normal tissues (*P* < 0.0001, paired t-test). Representative western blotting shows matched CRC and adjacent normal mucosa. **h** The relapse free survival (RFS) curves were plotted for CRC patients by Kaplan-Meier plotter. * *P* < 0.05, *** *P* < 0.001, and **** *P* < 0.0001 compared with the control group

Our previous proteogenomic work identified that NAMPT was upregulated in CRC compared to that in adjacent normal tissues, especially in CRC stages I and II [27] (Fig. 1a). Data obtained from the Cancer Genome Atlas (TCGA) also showed NAMPT mRNA expression levels in colon Adenocarcinoma (COAD) patient tissues were significantly higher than nontumorous tissues (Additional file 1: Figure S1A). Consistently, the dataset (Hong Colorectal) from Oncomine Cancer Microarray Database indicated that the expression levels of NAMPT were significantly reduced in advanced stage (M1 stage) of CRC patients compared with early stage (M0 stage) (Fig. 1b). GSE1323 dataset from Gene Expression Omnibus (GEO) indicated that NAMPT expression was significantly higher in primary CRC cell line SW480 than that in metastatic CRC cell line SW620 (Fig. 1c) [28]. Likewise, in silico analysis using the Oncomine Cancer Microarray Database revealed the significant upregulation of NAMPT expression levels in CRC tissues compared with those in nontumorous samples in different patient-derived datasets [29–31] (Fig. 1d). To further validate the expression level of NAMPT in CRC tissues, we performed real-time PCR, western blotting and IHC analyses in pairs of CRC and adjacent normal tissues. Tissue microarrays, including 50 pairs of paraffin-embedded CRC and adjacent normal mucosa, were used for the IHC analysis. The IHC staining score in the CRC group was significantly higher than that in the normal group (Fig. 1e). Real-time PCR and western blotting analysis of the 20 pairs of fresh-frozen CRC and adjacent normal mucosa were consistent with the IHC findings, indicating the markedly enhanced expression levels of NAMPT in CRC tissues compared to those in the adjacent normal mucosa (Fig. 1f and g). The Kaplan Meier plotter analysis showed upregulated level of NAMPT was associated with poorer relapse free survival (RFS) in CRC patients (Fig. 1h). However, Overall survival (OS) analysis based on NAMPT expression did not show any significant difference (Additional file 1: Figure S1B and Figure S1C). Together, the upregulated expression of NAMPT was identified in CRC tissues, and indicated unfavorable prognosis.

### Knockdown or pharmacological inhibition of NAMPT suppresses proliferation and promotes the apoptosis of CRC cells in vitro

**Fig. 2:**
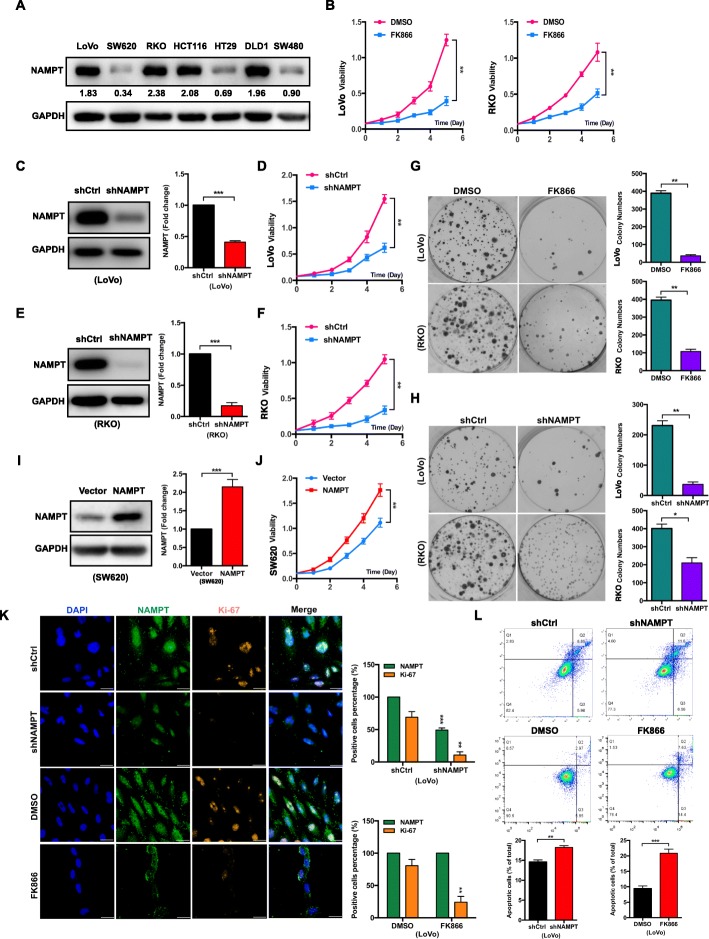
Inhibition of NAMPT decreases cell proliferation and induces cell apoptosis in CRC cells. **a** The protein expression levels of NAMPT in CRC cell lines were shown. **b** The viability of LoVo or RKO cells treated with 10 nM FK866 or 2% DMSO was analyzed using a CCK-8 assay. The data are presented as the mean ± SD of three independent experiments. Student’s t-test was used for statistical analysis. **c** The efficiency of NAMPT knockdown in LoVo cells was determined by western blot analysis. The data are presented as the mean ± SD of three independent experiments. Student’s t-test was used for statistical analysis. **d** The viability of LoVo cells that stably expressed shNAMPT or shCtrl was analyzed using a CCK-8 assay. The data are presented as the mean ± SD of three independent experiments. Student’s t-test was used for statistical analysis. **e** The efficiency of NAMPT knockdown in RKO cells was determined by western blot analysis. The data are presented as the mean ± SD of three independent experiments. Student’s t-test was used for statistical analysis. **f** The viability of RKO cells that stably expressed shNAMPT or shCtrl was analyzed using a CCK-8 assay. The data are presented as the mean ± SD of three independent experiments. Student’s t-test was used for statistical analysis. **g** Representative images of the colony formation assays using LoVo or RKO cells treated with 10 nM FK866 or 2% DMSO. The bar graphs show the quantification of the colony formation assay data. The data are presented as the mean ± SD of three independent experiments. Student’s t-test was used for statistical analysis. **h** Representative images of the colony formation assays using LoVo or RKO cells with shNAMPT or shCtrl. The bar graphs show the quantification of the colony formation assay data. The data are presented as the mean ± SD of three independent experiments. Student’s t-test was used for statistical analysis. **i** The efficiency of NAMPT overexpression in SW620 cells was determined by western blot analysis. The data are presented as the mean ± SD of three independent experiments. Student’s t-test was used for statistical analysis. **j** The viability of SW620 cells that stably overexpressed NAMPT or vector was analyzed using a CCK-8 assay. The data are presented as the mean ± SD of three independent experiments. Student’s t-test was used for statistical analysis. **k** The expressions of NAMPT and Ki-67 in shNAMPT LoVo, shCtrl LoVo cells, or in LoVo cells treated with DMSO or 10 nM FK866 were analyzed by immunofluorescence staining. The scale bar (white) indicates 20 μm. The quantification of NAMPT and Ki-67 expression are presented as the mean ± SD of three independent experiments. Student’s t-test was used for statistical analysis. **l** Representative images of the cell apoptosis assays in shNAMPT LoVo, shCtrl LoVo cells, or in LoVo cells treated with DMSO or 10 nM FK866. The bar graphs indicate the percentage of apoptotic cells. The data are presented as the mean ± SD of three independent experiments. Student’s t-test was used for statistical analysis. * *P* < 0.05, ** *P* < 0.01, and *** *P* < 0.001 compared with the control group

We first tested the protein expression levels of NAMPT in different CRC cell lines. NAMPT was abundant in certain CRC cell lines, such as LoVo, RKO, HCT116 and DLD1 cells (Fig. 2a). To clarify the role of NAMPT in CRC tumorigenesis, we constructed stable NAMPT knockdown (shNAMPT) cells from the LoVo and RKO cell lines and their respective negative control cells (shCtrl) [13] (Fig. 2c and e). CCK-8 analysis was performed to evaluate cell proliferation. The results showed that from day 3 to day 5, the mean absorbance of the shNAMPT groups was dramatically lower than that of the shCtrl groups in both LoVo and RKO cells (Fig. 2d and f). Consistently, the results of the monolayer colony formation assay showed that the knockdown of NAMPT retarded cell colony formation (Fig. 2h).

The NAMPT inhibitor, FK866 [15], was used to evaluate the effect on the growth of CRC cell lines. The CCK-8 results indicated that from day 3 to day 5, the mean absorbance of the FK866 group was significantly lower than that of the LoVo cell control group (Fig. 2b). The monolayer colony formation assay also showed that fewer colonies were formed in the FK866-treated LoVo group than the number of colonies that formed in the control group (Fig. 2g). Similar results were also observed in the RKO cells (Fig. 2b and g), SW620 cells (Additional file 1: Figure S2A and Figure S2B), and HCT116 cells settings (Additional file 1: Figure S2C). Intriguingly, we also generated stable NAMPT-overexpressing SW620 cells (Fig. 2i) and found the mean absorbance of the NAMPT overexpression group was significantly higher than that of the vector control group (Fig. 2j).

Using immunofluorescence staining, shNAMPT-LoVo cells displayed a lower rate of Ki-67-positive cells than the shCtrl-LoVo cells displayed, indicating a reduced proliferation ability (Fig. 2k). Similarly, FK866-treated LoVo cells showed a lower percentage of Ki-67-positive cells compared with that of the control cells (Fig. 2k). To determine whether the decreased proliferation was affected by apoptosis, we compared the apoptotic levels in the shNAMPT and shCtrl groups of LoVo cells. Flow cytometry results showed that the shNAMPT group harbored a significantly higher percentage of apoptotic cells than that of the shCtrl-LoVo cells (Fig. 2l). Flow cytometry results showed apoptotic level of FK866 group was significantly higher than that of DMSO groups (Fig. 2l).

Collectively, these results show that both the pharmacological inhibition and the knockdown of NAMPT inhibited proliferation and induced apoptosis in CRC cell lines.

### Inhibition of NAMPT does not have an impact on invasion and migration of CRC cells in vitro

To explore whether knockdown or pharmacological inhibition of NAMPT influences the migration capacity of CRC cells, we performed cell invasion and migration assays. However, NAMPT knockdown did not impair the invasion and migration capacity of LoVo cells. (Additional file 2: Figure S5A). NAMPT knockdown also did not impair the invasion and migration ability of HCT116 cells (Additional file 2: Figure S5B). Taken together, our results indicate that NAMPT inhibition does not influence invasion and migration abilities of colorectal cancer cells in vitro.

### NAMPT modulates CRC cell proliferation via the depletion of NAD^+^

**Fig. 3:**
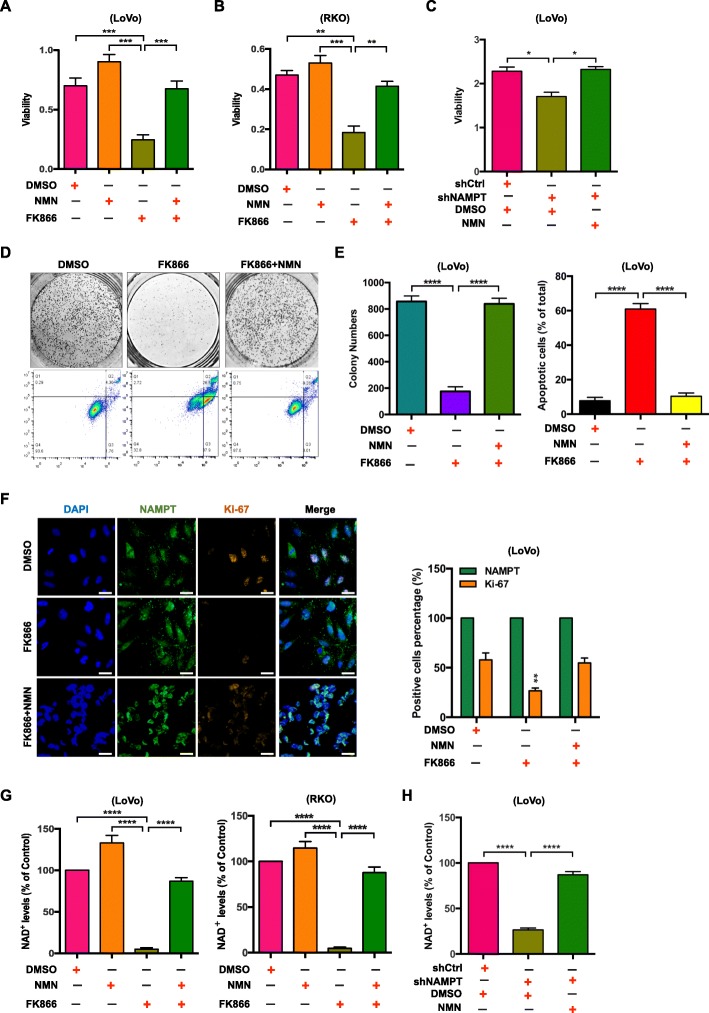
Pharmacological inhibition of NAMPT by FK866 or NAMPT knockdown reduces the NAD^+^ levels and cell viability of CRC cell lines. The viability assays of LoVo (**a**) or RKO (**b**) cells treated with 2% DMSO, FK866 (10 nM), NMN (100 μM), or NMN (100 μM) + FK866 (10 nM) for 3 days. The data are presented as the mean ± SD of three independent experiments. Student’s t-test was used for statistical analysis. **c** The viability of LoVo cells treated with shCtrl + 2% DMSO, shNAMPT + 2% DMSO, shNAMPT + NMN (100 μM) for 5 days. The data are presented as the mean ± SD of three independent experiments. Student’s t-test was used for statistical analysis. **d** Representative images of the colony formation and cell apoptosis assays using LoVo cells treated with 2% DMSO, FK866 (10 nM), or NMN (100 μM) + FK866 (10 nM). **e** The bar graphs show the quantification of the colony formation and cell apoptosis assay data. The data are presented as the mean ± SD of three independent experiments. Student’s t-test was used for statistical analysis. **f** The expressions of NAMPT and Ki-67 in LoVo cells treated with 2% DMSO, FK866 (10 nM), or NMN (100 μM) + FK866 (10 nM) for 3 days were analyzed by immunofluorescence staining. The scale bar (white) indicates 20 μm. The quantification of NAMPT and Ki-67 expression are presented as the mean ± SD of three independent experiments. Student’s t-test was used for statistical analysis. **g** The NAD^+^ levels of LoVo or RKO cells treated with 2% DMSO, FK866 (10 nM), NMN (100 μM), or NMN (100 μM) + FK866 (10 nM) for 3 days. The intracellular levels of NAD^+^ were measured using the NAD+/NADH Quantification Kit. The data are presented as the mean ± SD of three independent experiments. Student’s t-test was used for statistical analysis. **h** The NAD^+^ levels of LoVo cells treated with shCtrl + 2% DMSO, shNAMPT + 2% DMSO, shNAMPT + NMN (100 μM) for 5 days. The data are presented as the mean ± SD of three independent experiments. Student’s t-test was used for statistical analysis. * *P* < 0.05, ** *P* < 0.01, *** *P* < 0.001, and **** *P* < 0.0001 compared with the control group

To assess whether the inhibitory effects of shNAMPT and FK866 on CRC cell viability were due to the effects on NAD^+^ levels, we conducted rescue experiments by adding the intermediate of the NAD^+^ biosynthesis pathway back into the reaction. As expected, the cell viabilities could be rescued by the addition of the downstream intermediate of NAMPT (NMN, nicotinamide mononucleotide) both in LoVo and RKO cells (Fig. 3a and b), which were consistent with the NAD^+^ levels (Fig. 3g). The colony formation, apoptosis status and Ki-67 staining level could be rescued by NMN in FK866 treated LoVo cells (Fig. 3d, e and f). Similarly, the cell viability and the NAD^+^ level of shNAMPT-LoVo were rescued by adding NMN (Fig. 3c and h). NRK1 and NAPRT1 genes were previously reported to involve in NAD^+^ generation [12, 32]. The qRT-PCR results showed the expression levels of these two genes reduced upon treatment with FK866, which indicated NRK1 and NAPRT1 did not rescue NAMPT inhibition in CRC setting (Additional file 1: Figure S4). Therefore, we confirmed that NAMPT mediates CRC cell growth via the NAD^+^ level.

### Knockdown of NAMPT or FK866 inhibits the Wnt/β-catenin signaling pathway

**Fig. 4:**
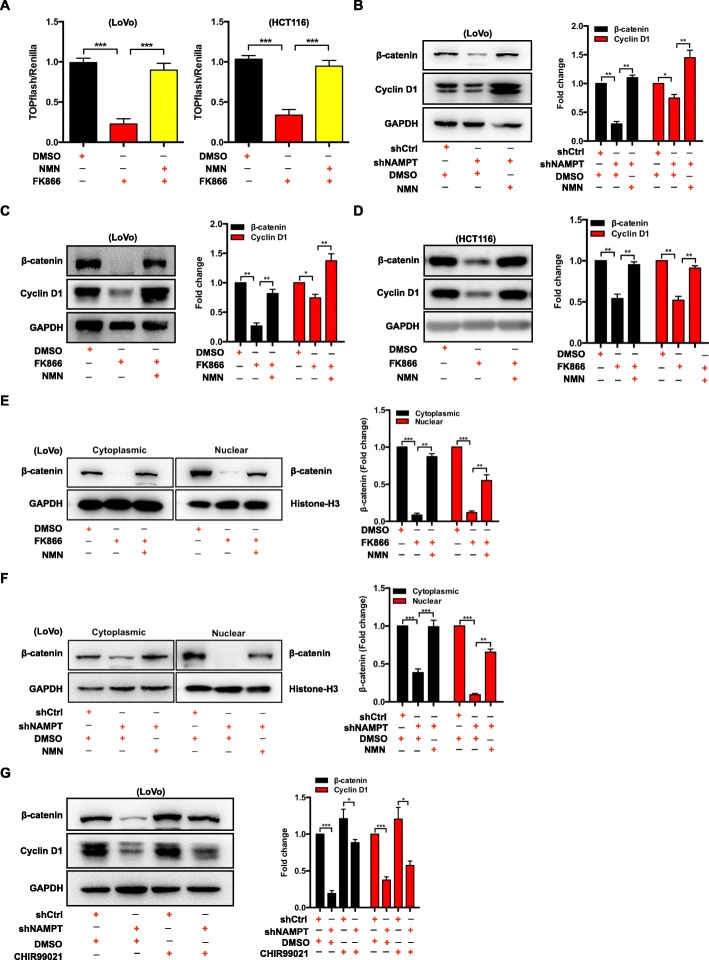
Modulation of the Wnt/β-catenin signaling pathway caused by NAMPT inhibition are NMN-dependent. **a** TOPFlash assay of LoVo or HCT116 cells treated with 2% DMSO, FK866 (10 nM), or NMN (100 μM) + FK866 (10 nM) for 2 days. The data are presented as the mean ± SD of three independent experiments. Student’s t-test was used for statistical analysis. **b** The expression of β-catenin and cyclin D1 in LoVo cells with shCtrl + 2% DMSO, shNAMPT + 2% DMSO, or shNAMPT + NMN (100 μM) was analyzed by western blotting. The data are presented as the mean ± SD of three independent experiments. Student’s t-test was used for statistical analysis. **c** Western blot analysis of whole cell lysates from LoVo cells exposed to 2% DMSO, FK866 (10 nM), or NMN (100 μM) + FK866 (10 nM) for 2 days showed the levels of β-catenin and cyclin D1. The data are presented as the mean ± SD of three independent experiments. Student’s t-test was used for statistical analysis. **d** Western blot analysis of whole cell lysates from HCT116 cells exposed to 2% DMSO, FK866 (10 nM), or NMN (100 μM) + FK866 (10 nM) for 2 days showed the levels of β-catenin and cyclin D1. The data are presented as the mean ± SD of three independent experiments. Student’s t-test was used for statistical analysis. **e** The cytoplasmic and nuclear expressions of β-catenin in LoVo cells treated with 2% DMSO, FK866 (10 nM), or NMN (100 μM) + FK866 (10 nM) for 2 days were analyzed by western blotting. The data are presented as the mean ± SD of three independent experiments. Student’s t-test was used for statistical analysis. **f** The cytoplasmic and nuclear expression of β-catenin in LoVo cells with shCtrl + 2% DMSO, shNAMPT + 2% DMSO, or shNAMPT + NMN (100 μM) was analyzed by western blotting. The data are presented as the mean ± SD of three independent experiments. Student’s t-test was used for statistical analysis. **g** The expression of β-catenin and cyclin D1 in LoVo cells with shCtrl + 2% DMSO, shNAMPT + 2% DMSO, shCtrl + CHIR99021 (100 ng/mL), or shNAMPT + CHIR99021 (100 ng/mL) was analyzed by western blotting. The data are presented as the mean ± SD of three independent experiments. Student’s t-test was used for statistical analysis. * *P* < 0.05, ** *P* < 0.01, and *** *P* < 0.001 compared with the control group

The Wnt/β-catenin signaling pathway plays a key role in regulation of CRC cell proliferation. Thus, we speculated that inhibition of NAMPT may lead to suppression of Wnt/β-catenin signaling. We tested the ability of NAMPT inhibition to suppress Wnt/β-catenin signaling using the TOP Flash reporter assay [33]. The TOP Flash reporter assay results showed that Wnt/β-catenin signaling was reduced by FK866, but could be rescued by adding NMN in LoVo, HCT116, and SW620 cells (Fig. 4a and Additional file 1: Figure S3A). The western blot results showed that in the FK866-treated LoVo or the shNAMPT-LoVo cells, the expression levels of β-catenin and Wnt/β-catenin target gene cyclin D1 were reduced; this suggests inhibition of Wnt/β-catenin signaling (Fig. 4 b and c). Similarly, the expression levels of β-catenin and cyclin D1 were reduced by FK866 and could be rescued by NMN in HCT116 cells (Fig. 4d). In addition, β-catenin nuclear translocation, which is a marker of Wnt signaling activation, was decreased in the FK866-treated and shNAMPT groups compared with that in the control group (Fig. 4e and f). Interestingly, by adding NMN to the FK866-treated LoVo or the shNAMPT-LoVo cells, nuclear β-catenin and cyclin D1 protein levels increased, thus, activating the Wnt/β-catenin signaling pathway (Fig. 4e and f). Utilization of CHIR-99021, the GSK3 inhibitor [34], resulted in higher β-catenin and cyclin D1 levels, thus activating Wnt/β-catenin signaling in LoVo cells (Fig. 4g). Interestingly, knockdown of NAMPT can inhibit the enhanced Wnt signaling induced by CHIR-99021 (Fig. 4g). These results strongly suggest that the knockdown of NAMPT or FK866 modulates the Wnt/β-catenin signaling pathway via NAD^+^.

### Knockdown of Axin can rescue the inhibitive effect on the proliferation of FK866

**Fig. 5:**
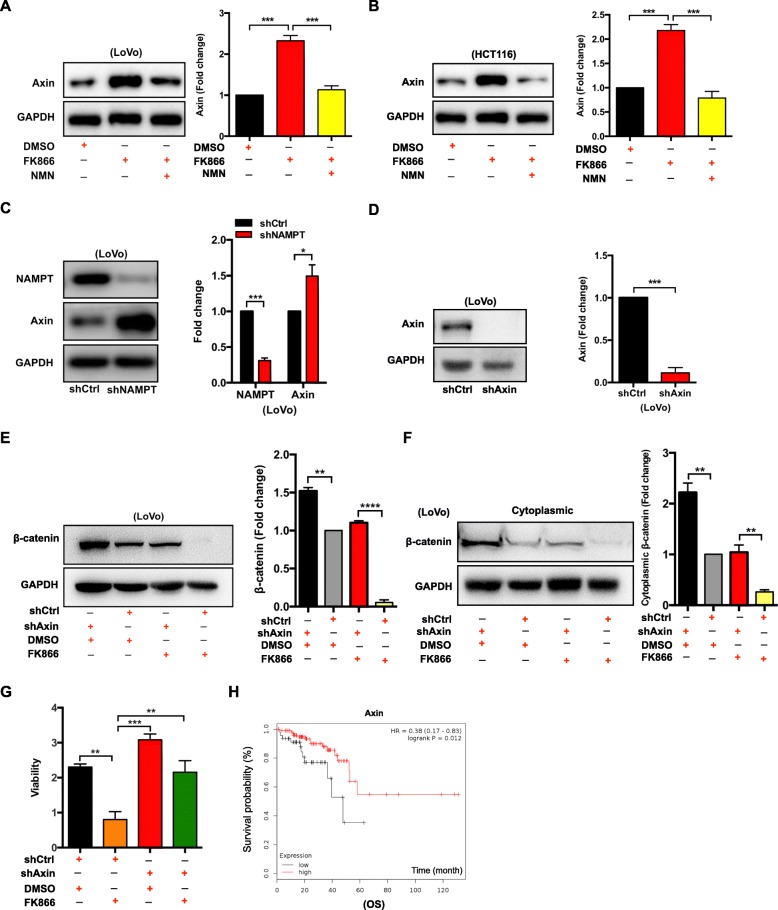
Knockdown of Axin can counteract the growth inhibition induced by NAMPT depletion via Wnt/β-catenin signaling activation. **a** Western blot analysis of whole cell lysates showed the protein levels of Axin from LoVo cells exposed to 2% DMSO, FK866 (10 nM), or NMN (100 μM) + FK866 (10 nM) for 2 days. The data are presented as the mean ± SD of three independent experiments. Student’s t-test was used for statistical analysis. **b** Western blot analysis of whole cell lysates showed the protein levels of Axin from HCT116 cells exposed to 2% DMSO, FK866 (10 nM), or NMN (100 μM) + FK866 (10 nM) for 2 days. The data are presented as the mean ± SD of three independent experiments. Student’s t-test was used for statistical analysis. **c** The expression of Axin in LoVo cells with shNAMPT or shCtrl was analyzed by western blotting. The data are presented as the mean ± SD of three independent experiments. Student’s t-test was used for statistical analysis. **d** The efficiency of Axin knockdown in LoVo cells was determined by western blot analysis. The data are presented as the mean ± SD of three independent experiments. Student’s t-test was used for statistical analysis. The expressions of β-catenin from whole cell lysates (**e**) and cytoplasmic lysates (**f**) of LoVo cells with shAxin + 2% DMSO, shCtrl + 2% DMSO, shAxin + FK866, or shCtrl +FK866 were analyzed by western blotting. Student’s t-test was used for statistical analysis. **g** The viability of LoVo cells with shCtrl + 2% DMSO, shCtrl + FK866, shAxin + 2% DMSO, or shAxin + FK866 was analyzed using a CCK-8 assay. Student’s t-test was used for statistical analysis. **h** Overall survival (OS) analysis of CRC patients based on Axin expression was analyzed by Kaplan-Meier plotter. * *P* < 0.05, ** *P* < 0.01, *** *P* < 0.001, and **** *P* < 0.0001 compared with the control group

The western blot results also indicated that in the FK866-treated LoVo or the shNAMPT-LoVo cells, the expression levels of Axin increased (Fig. 5a and c). Consistently, the expression levels of Axin increased in FK866-treated HCT116 cells (Fig. 5b). Moreover, the expression level of Axin could be reduced by NMN in FK866-treated LoVo and HCT116 cells (Fig.5a and b). To further investigate how the Wnt/β-catenin signaling pathway is involved in this process, we established stable shAxin-LoVo cells to perform a rescue assay (Fig. 5d). Western blotting analysis showed that in the shAxin-LoVo+FK866 group, β-catenin expressions from both whole cell lysis and cytoplasmic cell lysis were upregulated compared with that in the shCtrl-LoVo+FK866 group, indicating that Axin knockdown counteracted the regulatory effect of FK866 on Wnt/β-catenin signaling (Fig. 5e and f). Intriguingly, the CCK-8 assay indicated that the shAxin-LoVo+FK866 cells proliferated much more rapidly than the shCtrl-LoVo+FK866 cells (Fig. 5g). In clinical setting, overexpression of Axin also indicated better OS in CRC patients (Fig. 5h). These results suggest that the knockdown of Axin mitigates the inhibitory role of FK866 on cell proliferation via enhanced Wnt/β-catenin signaling, and Axin expression is a favorable prognostic indicator in CRC.

### Knockdown of NAMPT inhibits CRC proliferation in vivo

**Fig. 6:**
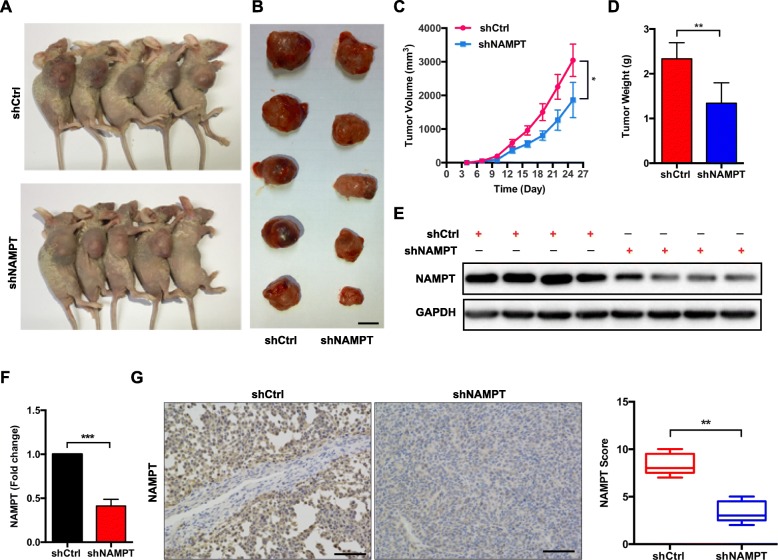
Knockdown of NAMPT suppresses CRC cell growth in vivo. **a**-**d** The effect of NAMPT expression on in vivo tumorigenicity was evaluated using a xenograft nude mouse model. **a** Tumors formed in nude mice; **b** dissected subcutaneous tumors, scale bars: 10 mm; **c** curves of tumor growth; **d** tumor weight; number = five mice/group. The error bars indicate the SD. Student’s t-test was used for statistical analysis. **e** The protein expression levels of NAMPT in xenograft tumors were analyzed by western blotting. **f** Quantification of the protein expression levels of NAMPT in xenograft tumors. Student’s t-test was used for statistical analysis. **g** Representative images of IHC staining for NAMPT in the xenografts. The box graphs show the quantification of IHC staining. Scale bars: 50 μm. Student’s t-test was used for statistical analysis. * *P* < 0.05, ** *P* < 0.01, and *** *P* < 0.001 compared with the control group

To further confirm the impact of NAMPT on tumor growth in vivo, tumor xenograft mouse models were constructed by subcutaneously injecting shNAMPT-LoVo cells or shCtrl-LoVo cells (5 × 10^6^ cells per mouse). All mice were euthanized, and the subcutaneous xenograft tumors were dissected and weighed 25 days after the injection (Fig. 6a and b). The xenograft tumor growth curves and the average tumor weight of the shNAMPT-LoVo and shCtrl-LoVo cell groups suggested that the knockdown of NAMPT inhibited CRC proliferation in vivo (Fig. 6c and d). The IHC analysis indicated that the NAMPT expression levels in the xenograft tumors in the shNAMPT-LoVo group were significantly lower than those in the shCtrl-LoVo group (Fig. 6g). The western blot results also consistently showed a significant reduction of NAMPT in the shNAMPT-LoVo group compared with that in the shCtrl-LoVo group (Fig. 6e and f).

## Discussion

**Fig. 7:**
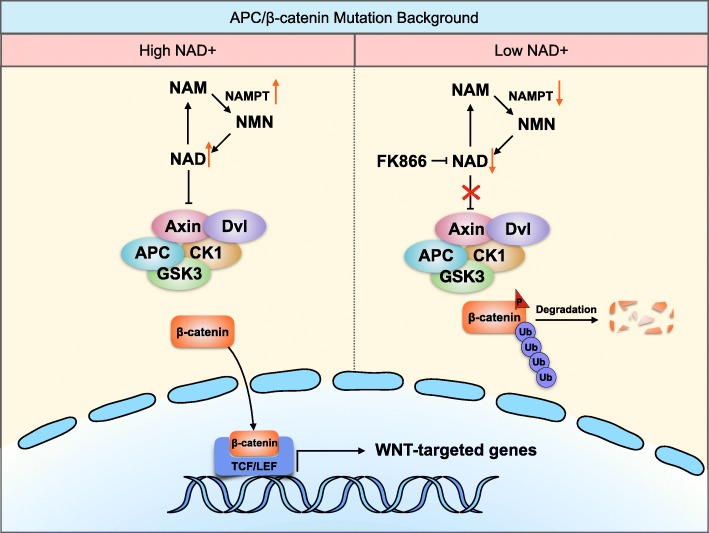
Model of NAMPT’s role in the proliferation of human CRC cells through the regulation of Wnt/β-catenin signaling. A high level of NAMPT (left panel) yields more NAD^+^ and induces downregulation of Axin and the nuclear translocation of β-catenin, thereby leading to the activation of Wnt/β-catenin signaling and the increased growth of CRC cells. However, when NAMPT is inhibited by genetic or pharmacological methods (right panel), the NAD^+^ level is reduced and Axin is upregulated. Then, β-catenin is degraded, and the Wnt/β-catenin pathway is suppressed, resulting in the inhibition of cancer cell proliferation

In this study, we highlighted a novel mechanism by which high NAD^+^ levels promote the proliferation of CRC cells by further activating the Wnt/β-catenin signaling pathway (Fig. 7). NAD^+^ deprivation by the pharmacological inhibitor FK866 or by the knockdown of NAMPT could suppress the growth of CRC cells. The low NAD^+^ levels promoted β-catenin degradation through upregulated Axin expressions. Restoring the NAD^+^ levels by utilizing the NAD^+^ intermediate NMN successfully counteracted the growth inhibitory effect of FK866 or shNAMPT in CRC cells by activating Wnt/β-catenin signaling. In addition, the knockdown of Axin also restored the β-catenin protein levels and the cell viability of CRC cells treated with FK866; this further validates that the inhibition of NAMPT modulates Wnt/β-catenin signaling. Our findings suggest that NAMPT is a novel target for Wnt inhibition by promoting β-catenin degradation through increasing Axin levels.

NAD^+^ is a fundamental molecule that participates in a multitude of vital cellular processes, including metabolism, inflammation, DNA repair, immune response, cell cycle control, autophagy and cell death [11, 14, 35–37]. Since a sufficient NAD^+^ supply is critical for cell viability, NAMPT plays an important role in facilitating cell proliferation. Upregulated NAMPT boosts the initiation and progression of various cancers, and the interference of NAMPT by chemical inhibitors or by genetic modification decreases the survival of cancer cells [14, 38]. Our data are in accordance with the data of previous studies showing that the inhibition of NAMPT has a negative correlation with the growth of CRC cells.

Recently, some studies have indicated that NAMPT acts as a regulator of cancer invasion and metastasis [5, 39, 40], which is one of the hallmarks of malignant cancer cells [41]. However, the current understanding of the role that NAMPT plays in invasion and metastasis remains unclear. The overexpression of NAMPT could significantly enhance the abilities of invasion and migration in osteosarcoma and glioma cells [5, 39, 40]; however, some studies draw the opposite conclusion that the knockdown of NAMPT promotes metastatic aggressiveness in breast cancer cells [42]. Intriguingly, NAMPT-induced epithelial-to-mesenchymal transition has been shown to be modulated by secreted NAMPT and to be independent of FK866 and of NAD^+^ intermediate treatment [43]. Therefore, the positive correlation between NAMPT overexpression and the migration capacity in some studies might be due to the increased secretion of NAMPT [39]. Our results indicate that the knockdown of NAMPT failed to impair the tendency of invasion and migration in both HCT116 and LoVo cells, but we do not know whether the overexpression of NAMPT in this setting would influence the migration ability. Further investigation is warranted to determine the function of NAMPT in the invasion and migration of CRC cells.

The most important finding from this study is the discovery of the linkage between NAMPT and Axin. Axin is an important concentration-limiting factor for the destruction complex stability [44–46], and the overexpression of Axin can lead to β-catenin degradation in cell lines with truncated APC [46–48]. To our knowledge, this is the first study to elucidate whether NAD^+^ exerts a positive effect on the growth of cancer cells via the Wnt/β-catenin pathway. The Axin protein level is inhibited via poly(ADP-ribosyl)ation (PARsylation) by tankyrases (TNKS) [46], which control the ubiquitylation of Axin by RNF146 and proteasomal degradation [49, 50]. Several TNKS inhibitors have been identified to stabilize Axin by preventing PARsylation and ubiquitylation activities, thus promoting β-catenin degradation [46, 51, 52]. Given that NAD^+^ is the obligatory cosubstrate of TNKS, the increased NAMPT-mediated production of NAD^+^ could boost TNKS catalysis in β cells [53]. However, it is unknown whether the NAD^+^ level has an impact on increasing Axin protein level by TNKS inhibition in cancer cells and whether it is sufficient to impact cancer proliferation in the APC-mutant cancer setting. Our study highlights that NAMPT inhibition could retard the growth of APC-truncated CRC via Axin overexpression.

NAMPT was reported to be upregulated in several malignant cancers, including prostate, breast, melanoma, colon, and hematologic malignancies [5, 9, 13, 38, 54–58]. In bladder cancer, a high serum NAMPT level was found to serve as a biomarker and an independent prognostic indicator [59]. In BRAF-mutated melanoma, the high expression of NAMPT is associated with targeted therapy resistance, and NAMPT is a therapeutic target for this subset of patients [16, 60]. Our results indicated that the overexpression of NAMPT was detected in CRC tissues, especially in stages I and II, and was correlated with worse survival outcomes in CRC patients. A variety of phase I and II clinical trials included the use of NAMPT inhibitors, and many researchers are exploring a novel inhibitor design for NAMPT-targeted treatment [61–63]. Therefore, NAMPT is a promising biomarker and target for treatment in early stage CRC.

In conclusion, we show that NAMPT is overexpressed in CRC and boosts cell proliferation, which occurs via the activation of the Wnt/β-catenin pathway. This novel molecular mechanism may serve as a promising biomarker and treatment target for CRC. Since the alteration of the NAD^+^ metabolism occurs in a variety of cancers, the therapeutic options in NAD^+^ metabolism could also be utilized in the treatments of other Wnt-dependent cancers.

## Conclusions

In summary, we demonstrate that high level of NAMPT expression enhances NAD^+^ production and ultimately leads to increased proliferation in CRC cells by activating the Wnt/β-catenin pathway. We propose that NAMPT serves as a potential biomarker and therapeutic target for patients with CRC.

## Supplementary information

**Additional file 1: Figure S1.** NAMPT mRNA expression analysis in colorectal cancer (CRC). (A) NAMPT mRNA levels in colon Adenocarcinoma (COAD) patient tissues compared to nontumorous tissues. Data were obtained from the Cancer Genome Atlas (TCGA). (B) The prognostic value of NAMPT mRNA expression in CRC patients for overall survival (OS) was analyzed by Kaplan-Meier plotter. (C) The prognostic value of NAMPT mRNA expression in CRC patients for overall survival (OS) was analyzed by using dataset GSE17536. **** *P* < 0.0001 compared with the control group. **Figure S2.** FK866 inhibits the proliferation of SW620 and HCT116 cells in vitro. (A) The viability of SW620 cells treated with FK866 (10 nM) or 2% DMSO was analyzed using a CCK-8 assay. The data are presented as the mean ± SD of three independent experiments. Student’s t-test was used for statistical analysis. (B) Representative images of the colony formation assays using SW620 cells treated with FK866 (10 nM) or 2% DMSO. The bar graphs show the quantification of the colony formation assay data. The data are presented as the mean ± SD of three independent experiments. Student’s t-test was used for statistical analysis. (C) The viability of HCT116 cells treated with FK866 (10 nM) or 2% DMSO was analyzed using a CCK-8 assay. The data are presented as the mean ± SD of three independent experiments. Student’s t-test was used for statistical analysis. * *P* < 0.05, and *** *P* < 0.001 compared with the control group. **Figure S3.** FK866 inhibits Wnt/β-catenin signaling. (A) TOPFlash assay of SW620 cells treated with 2% DMSO, FK866 (10 nM), or NMN (100 μM) + FK866 (10 nM) for 2 days. The data are presented as the mean ± SD of three independent experiments. Student’s t-test was used for statistical analysis. *** *P* < 0.001 compared with the control group. **Figure S4.** Inhibition of FK866 is not rescued by NRK1 or NAPRT1. (A) The mRNA expression levels (qRT-PCR analysis) of NRK1 and NAPRT1 in LoVo cells treated with 2% DMSO or FK866 (10 nM). The data are presented as the mean ± SD of three independent experiments. Student’s t-test was used for statistical analysis. * *P* < 0.05 compared with the control group.

**Additional file 2: Figure S5.** shNAMPT does not influence the migration and invasion of colorectal cancer cells in vitro. (A) Transwell migration and invasion assays using LoVo cells with shNAMPT or shCtrl. The quantification of migration or invasion cell numbers are presented as the mean ± SD of three independent experiments. Student’s t-test was used for statistical analysis. (B) Transwell migration and invasion assays using HCT116 cells with shNAMPT or shCtrl. The quantification of migration or invasion cell numbers are presented as the mean ± SD of three independent experiments. Student’s t-test was used for statistical analysis. n.s. indicates no significant difference.

## Data Availability

The datasets used and/or analyzed during the current study are available from the corresponding author on reasonable request.

## Abbreviations

APC: Adenomatous polyposis coli gene product
COAD: Colon adenocarcinoma
CRC: Colorectal cancer
GSK3: Glycogen synthase kinase 3
IHC: Immunohistochemistry
NA: Nicotinic acid
NAD^+^: Nicotinamide adenine dinucleotide
NAM: Nicotinamide
NAMPT: Nicotinamide phosphoribosyltransferase
NMN: Nicotinamide mononucleotide
NR: NAM riboside
PARsylation: Poly(ADP-ribosyl)ation
PRPP: 5-phosphoribosyl-1-pyrophosphate
TCGA: Cancer Genome Atlas
TMA: Tissue microarray
TNKS: Tankyrases
