# Supplementary figures and images for: Targeting the NAD+ salvage pathway suppresses APC mutation-driven colorectal cancer growth and Wnt/β-catenin signaling via increasing Axin level
Source: Cell Commun Signal. 2020 Jan 31;18:16. doi: 10.1186/s12964-020-0513-5 (PMC6995173; doi:10.1186/s12964-020-0513-5)

**Fig. S1**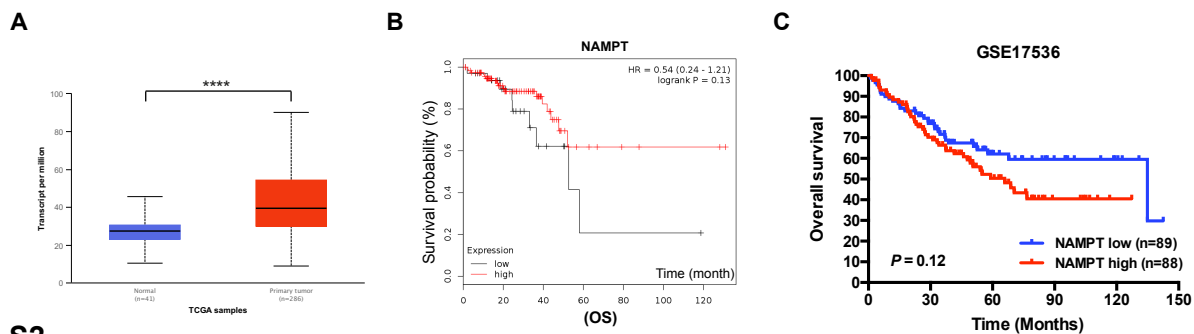**Fig. S2**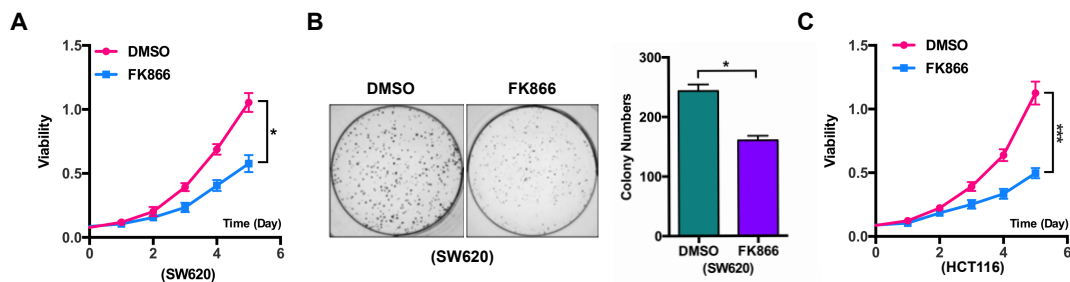**Fig. S3**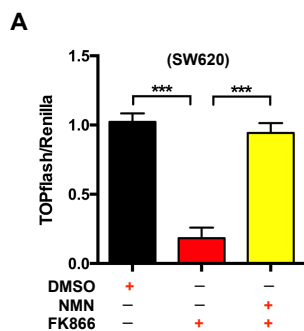**Fig. S4**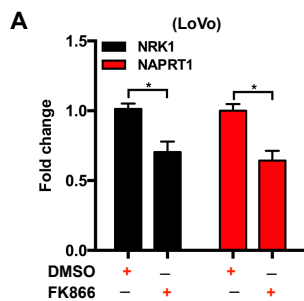

Supplement: Supplementary file 1 — Additional file 1: Figure S1. NAMPT mRNA expression analysis in colorectal cancer (CRC). (A) NAMPT mRNA levels in colon Adenocarcinoma (COAD) patient tissues compared to nontumorous tissues. Data were obtained from the Cancer Genome Atlas (TCGA). (B) The prognostic value of NAMPT mRNA expression in CRC patients for overall survival (OS) was analyzed by Kaplan-Meier plotter. (C) The prognostic value of NAMPT mRNA expression in CRC patients for overall survival (OS) was analyzed by using dataset GSE17536. **** P < 0.0001 compared with the control group. Figure S2. FK866 inhibits the proliferation of SW620 and HCT116 cells in vitro. (A) The viability of SW620 cells treated with FK866 (10 nM) or 2% DMSO was analyzed using a CCK-8 assay. The data are presented as the mean ± SD of three independent experiments. Student’s t-test was used for statistical analysis. (B) Representative images of the colony formation assays using SW620 cells treated with FK866 (10 nM) or 2% DMSO. The bar graphs show the quantification of the colony formation assay data. The data are presented as the mean ± SD of three independent experiments. Student’s t-test was used for statistical analysis. (C) The viability of HCT116 cells treated with FK866 (10 nM) or 2% DMSO was analyzed using a CCK-8 assay. The data are presented as the mean ± SD of three independent experiments. Student’s t-test was used for statistical analysis. * P < 0.05, and *** P < 0.001 compared with the control group. Figure S3. FK866 inhibits Wnt/β-catenin signaling. (A) TOPFlash assay of SW620 cells treated with 2% DMSO, FK866 (10 nM), or NMN (100 μM) + FK866 (10 nM) for 2 days. The data are presented as the mean ± SD of three independent experiments. Student’s t-test was used for statistical analysis. *** P < 0.001 compared with the control group. Figure S4. Inhibition of FK866 is not rescued by NRK1 or NAPRT1. (A) The mRNA expression levels (qRT-PCR analysis) of NRK1 and NAPRT1 in LoVo cells treated with 2% DMSO or FK866 (10 [file 12964_2020_513_MOESM1_ESM.pdf]

**Fig. S5**

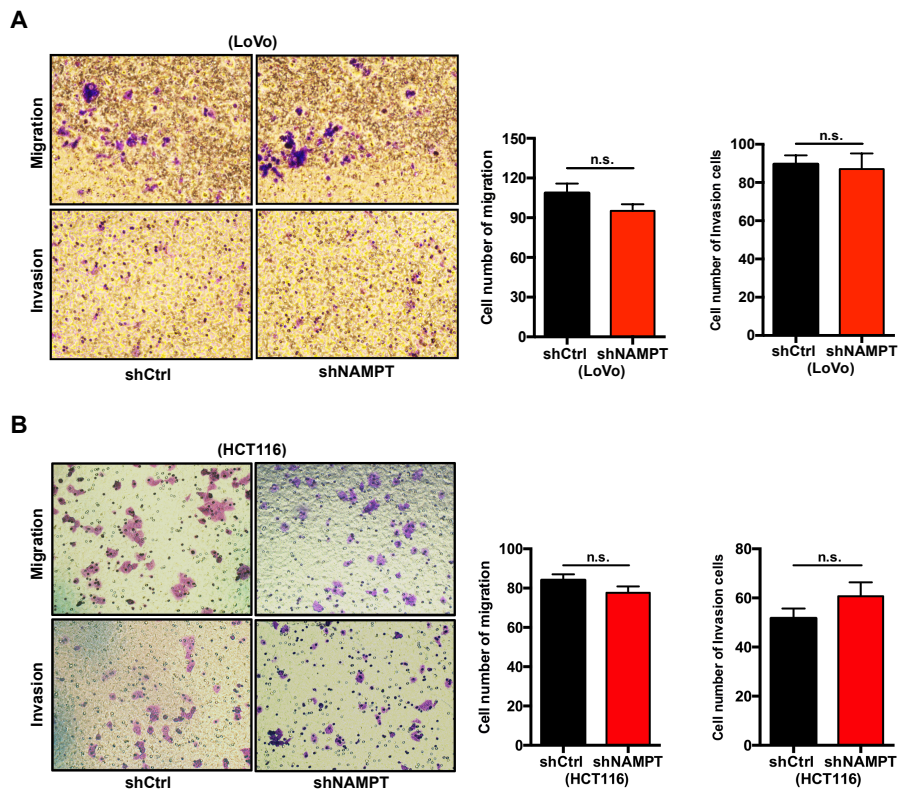

Supplement: Supplementary file 2 — Additional file 2: Figure S5. shNAMPT does not influence the migration and invasion of colorectal cancer cells in vitro. (A) Transwell migration and invasion assays using LoVo cells with shNAMPT or shCtrl. The quantification of migration or invasion cell numbers are presented as the mean ± SD of three independent experiments. Student’s t-test was used for statistical analysis. (B) Transwell migration and invasion assays using HCT116 cells with shNAMPT or shCtrl. The quantification of migration or invasion cell numbers are presented as the mean ± SD of three independent experiments. Student’s t-test was used for statistical analysis. n.s. indicates no significant difference. [file 12964_2020_513_MOESM2_ESM.pdf]
